# Correction to “Use of Coaching and Technology to Improve Blood Pressure Control in Black Women With Hypertension: Pilot Randomized Controlled Trial Study”

**DOI:** 10.1111/jch.70085

**Published:** 2025-10-21

W. M. Abel, J. T. Efird, P. B. Crane, K. C. Ferdinand, C. G. Foy, and M. J. DeHaven, “Use of Coaching and Technology to Improve Blood Pressure Control in Black Women With Hypertension: Pilot Randomized Controlled Trial Study,” *Journal of Clinical Hypertension* 25, no. 1 (2023): 95–105, https://doi.org/10.1111/jch.14617.

An incorrect trial registration number appeared in the published article. The correct ClinicalTrials.gov identifier is NCT03577990.

We apologize for this error.

